# Obstetrics

**Published:** 1875-10

**Authors:** 


### Summary of Progress in the Medical Sciences.

### I. Obstetrics.

1. *Hæmorrhagic Cerebral Tumor—Epilepsy—Post-Mortem Cæsarean Section.* Pinard. (*Le Progrès Medical,* July 10, 1875.)

The author reports the case of a primipara, 22 years of age; plunged in profound prostration, half somnolent, with closed eyes; unable to give coherent answers to questions; sensibility was intact; motility, normal on the right side, and enfeebled in both the upper and lower left extremities. The tongue was projected to the left; pupils widely dilated; no strabismus, nor irregularity of nostrils or eyelids. Integument pallid; some embonpoint; abdomen distended as in pregnancy between sixth and seventh month. Fœtus active, with heart sounds distinct in left hypogastrium; cervix uteri of due length; quite flaccid, and having closed orifice. No sugar nor albumen detected in a scanty, lemon-colored urine. Respirations regular, slightly stertorous—twenty-four to the minute; pulse 80; axillary temperature, 37.2° C.

The very exceptional absence of albumen in the urine, and the character of the repeated epileptic seizures which soon followed, led to the diagnosis of a cerebral tumor. The convulsions rapidly increased in frequency, from one or two in the day, until the last hours of the patient were passed in one convulsive spasm, accompanied by incontinence of urine and fæces, and followed by coma and death. Pulsations of the fœtal heart being then audible, Dr. Pinard performed Cæsarean section; and extracted a child apparently moribund, but whose heart continued to beat regularly. Respiration followed after insufflation for fifteen minutes. The child, however, did not survive more than three hours.

Post-mortem examination of the mother disclosed scirrhous induration of a voluminous pineal gland, with disappearance of the posterior apophyses of the sella turcica. All the ventricles were more or less distended with sero-sanguinolent fluid. The right lateral ventricle contained also clots—together, forming a mass as large as an orange—while a mamelonnated tumor projected from its lateral and superior wall. There was considerable softening of the adjacent cerebral substance. A second smaller and similar tumor was also found in the lateral ventricle.

These tumors were found to contain blood, enveloped in a laminated membrane, whose lamellæ became finer from periphery to centre. The latter were found to be made up of a delicate vascular stroma, best organized in the vicinity of the clot, and having, at certain points, undergone fatty degeneration.

The points of interest in this case were, the differential diagnosis between puerperal eclampsia and the influence of a cerebral tumor; the persistence of fœtal life, with rhythmic and forcible pulsations of the heart during a long series of epileptic convulsions; and the complete post-mortem contraction of the uterus after the removal of its contents. The judicious obstetrician of the Maternité had operated precisely as if the woman were living, and when he exhibited the womb and its appendages to the school of midwives connected with the hospital, he was enabled to call their attention to the form and volume of the organ, which did not differ from that of a woman delivered in the natural method.

2. *Vesical Ecchymoses in the Newly Born.* PARROT. (*Le Progres Medic.*, June 10.)

The reporter exhibited before the Anatomical Society some pathological specimens removed from the body of a newly born child who had presented symptoms of that form of œdema, improperly designated scleremia. It had suffered from the kind of black vomit often observed in cancerous cases. There were, post-mortem, evidences of ecchymatic, non-ulcerative erosions in the œsophagus and stomach and upon the surface of the kidneys, as well as within the intestines (particularly the rectum). But especial attention was called to those in the bladder, which were rather small tumors, as large as grape seeds, produced by submucous hæmorrhages.

The reporter estimated that these vesical ecchymoses, of greater or less projection, were to be found in seven or eight of every ten cases of œdema of the newly born.

3. *Influence of the Nervous System upon Phenomena of Pregnancy and Parturition.* GOLTZ and FREUSBURG. (*Archiv. fur Phys.*, ix. 1874.) Note by Fritsch. (*Jahrbücher* 1875; *Phil. Med. Times.*)

Section of spinal medulla and left sciatic nerve in a bitch, nine months old, was succeeded by recovery (excepting the paralysis) and increase in weight. Pregnancy occurred in four months, with birth of strong living pup, cleaned by licking. The placenta was devoured. Two others, dead-born, were similarly treated. Rhythmical flexion and extension of posterior extremities were coordinate with labor pains. There was also contraction of intra-pelvic muscles, and uncontracted vagina. Peritonitis and death resulted from vaginal perforation. Segments of the medulla were found one centimetre separate —the lower of normal size.

Fritsch considered that the integrity of the ovaries, explained the going into heat, at which time the male, previously refused, was accepted. Presence or absence of ovaries, therefore, affected the brain centres ; and the sympathetic fibres must have joined the cord above the site of section (lumbar region). It is probable that, during heat, characteristic matters enter the blood and thus act upon the brain. Impregnation without sensation is possible, since women conceive when stunned. The development of distant organs often depends upon presence of essential genitalia. Körner asserts the medullary origin of uterine motor fibres about the last dorsal vertebra. Thence they descend along the aorta. These, in the experiment, would have been uninjured. Probably the entire parturient act does not depend upon the brain, as other fibres originate about the third or fourth lumbar vertebræ. In this case, action of the external muscles could not have originated in impulses from the brain, but from a nervous centre in the lumbar region of the cord. This centre may extend over the whole cord, so that, when divided, each segment may be influential so long as there is integrity of centrifugal fibres. The third and fourth cervical vertebræ were fractured in a primipara, aged 24, between the sixth and seventh month of pregnancy, with consequent paralysis of sensation and motion below seat of injury, but labor-pains, unfelt by her, still expelled a dead-born child. This case also tends to show the existence of an independent centre in the cord.

4. *Report of the Board of Health of the City and Port of Philadelphia for the Year* 1874.

This excedingly valuable Report, under the head of Births, gives some interesting statistics, with deductions of scientific import. The birth rate to 1,000 living persons, was, in the United States, (1870), 28.86 ; England, (1861—1870), 35.2 ; France, (1861—1870), 26.2 ; Prussia, (1861—1870), 39.6 ; Austria, (1861—1870), 39.8 ; New

York, (1874), 24.46; Philadelphia, (1870—1873,) 24.88; Chicago, (1874), 22.87; Boston, (1874), 31.18. Imperfect registration laws and returns account, probably, for some of the differences.

From 1861 to 1873, 100 males were born alive to 109.4 females; and 100 of the former to 136.5 of the latter, dead born. In 1874 the corresponding figures were, 100 to 110.7, and 100 to 135.

During the past fourteen years, the births in Philadelphia were quite evenly distributed in the different months, with the exception of April, May and June. The decrease in these months is attributed to influences operating in the preceding summer and in the commencement of autumn.

Dr. Emerson obtained similar results from examination of the birth statistics in Philadelphia for the ten years 1821—1830. Dr. Villermé, of Paris, who has collected and analyzed a very large number of births, calls attention to a decrease in the birth rate during these same months. Both writers attribute these results to the influence of the seasons upon conception.

From 1861 to 1874, the greatest number of *conceptions* occurred in April; then, in January, March, December and November; the least number in July; next, August, September and May. Almost without exception, the coldest months were the most prolific, and the warmest the least so. In Montpellier, France, February was the most prolific month of the year; then followed in order, April, May, March and January. The smallest number of conceptions took place in September; next in August, and then in July and October. In both places the most unfavorable months for reproduction were the same, viz.: July, August and September, though in Philadelphia the order by months is reversed.

Milne is of opinion that if the disturbing element of marriage were as accurately regulated as that of births, the influence of the seasons upon conception would be still more manifest, and that then the maximum of conceptions would be found to occur in midsummer.

Baker, of Michigan, from his statistics, deduces: "1. That the uniform proportion of births by seasons, is, in the majority of months, influenced by the number of marriages. 2. That, for the reason that the excess or deficit in the number of births, compared with the average number, is larger than the excess or deficit of marriages for the months in which the conceptions occurred, compared with the average number of marriages, it would appear that the marriage-rate is not, to say the least, the only cause of the uniform excess or deficiency of births at different seasons of the year. 3. Taking the two foregoing propositions together, it seems that both the marriage and birth-rate are, to some extent, dependent upon the same cause, which, although not manifest here, it is altogether probable is a physiological one, and directly connected with climate or seasons of the year."*

In Philadelphia, from the statistics for thirteen years, (1862—1874), it appears that of the seven months in which more than an average number of conceptions occurred, there were four months having more than an average number of marriages; but, in the remaining five months, having less than an average number of conceptions, there were four months having less than an average number of marriages.

5. *Hydrate of Chloral in Obstetric Practice.* CHIARLEONI. (*Gazet. Med. Ital. Lomb.; Brit. Med. and Surg. Jour.*, Aug. 19.)

The usual formula was: six grammes of chloral, sixty of syrup, and one hundred of water. A spoonful of this was given till the desired effect was produced, except when larger doses were necessary, when four grammes of chloral were divided into two enemata, given with an hour's interval.

There were four classes of patients: 1st. Irritable, hysterical, nervous or apprehensive (primiparous) women, with feeble, interrupted and ineffectual pains. Result: effectual uterine contraction, sleep, diminution of pain,

* Fourth Registration Report, Michigan.

and shortening of period of labor. 2nd. Patients with albuminuria, the remedy preventing convulsive action. 3rd. Cases in which the drug was administered to render operations easy and less painful. In the greater number of cases, however, the chloral was given after the termination of labor, to promote rest or sleep.

These experiments indicated that the treatment did not tend to retard the progress of labor or injure the child.

In from one to five or more hours after exhibition of the remedy, according to the dose and the peculiar circumstances of the case, the desired results had been accomplished. Occasionally talkativeness and hiliarity were induced.

6. *Procedure in Right Occipito-posterior Position.* PROF. F. BARKER. (*Med. Record*, June 19.)

The Professor at first attempted to rotate the occiput into the anterior position through the vagina, but was foiled in this, as the occiput would rotate back into the hollow of the sacrum, as soon as the hand was removed. The woman was then carried profoundly under the influence of chloroform, the left hand introduced, and, with a great deal of effort, the head was pushed up out of the pelvic cavity. Then, with the right hand upon the abdomen, rotation of the trunk was effected, so that the occiput was brought around. Then the head was pressed under the symphysis pubis, and delivery of a living child accomplished without perineal rupture. He was quite sure that by this operation he saved the perineum and also the life of the child.

7. *Gastro-Elytrotomy.* THOMAS. (*Am. Jour. of Obstetrics*, Aug., 1875.)

In the case of a woman, moribund and near the end of pregnancy, the reporter, instead of performing Cæsarean section, made an incision in the inguinal region parallel to Poupart's ligament, down to the peritoneum. He then pushed up the vagina with the sound, cut down upon it, and enlarged the opening with the scissors. The hand was then passed through the large incision into the cervix, which had been dilated with Barnes' dilator. The feet of the child were seized, version was performed, and the child extracted alive—the latter surviving the operation for several days, dying eventually from causes unconnected with the operation. The woman was pulseless, and died in four hours. The time occupied in performing the operation was short—not longer than that required for Cæsarean section. The reporter believed he could perform the operation and deliver the child in five minutes. He would not always prefer this method to Cæsarean section, and was fully aware of the incidental danger of cellulitis, septic infection and hæmorrhage, especially in the vascular condition of the vagina during pregnancy. But he was inclined to think the operation had a future. So far as he was aware, but one operation of the kind had since been performed—that by Dr. Skene, of Brooklyn. The child had been previously perforated and the woman exhausted. Death occurred in seven hours. He thought that drainage through the vagina would diminish the chances of septicæmia. From the description of the operation it would appear to be a difficult one; but, in point of fact, it is surprisingly easy in every detail.

8. *Vicarious Menstruation.* DR. J. N. UPTHUR, Richmond, Va. (*Virginia Med. Monthly*, Sept., 1875.)

The case reported was a women 18 years old, who commenced menstruating at 13 and continued it 2 years regularly thereafter. Periods attended with pain. "For past 3 years has suffered from amenorrhœa, but for the past 10 months, has had, every 4 weeks, a vicarious discharge of blood from the stomach, attended by cough, nausea, hæmatemesis, rachialgia, tympanitis and sense of weight at hypogastrium." Under iron, strychnine and ergot she soon menstruated normally.

